# Augmented Reality Surgical Navigation System for External Ventricular Drain

**DOI:** 10.3390/healthcare10101815

**Published:** 2022-09-21

**Authors:** Shin-Yan Chiou, Zhi-Yue Zhang, Hao-Li Liu, Jiun-Lin Yan, Kuo-Chen Wei, Pin-Yuan Chen

**Affiliations:** 1 Department of Electrical Engineering, College of Engineering, Chang Gung University, Kwei-Shan, Taoyuan 333, Taiwan; 2 Department of Nuclear Medicine, Linkou Chang Gung Memorial Hospital, Taoyuan 333, Taiwan; 3 Department of Neurosurgery, Keelung Chang Gung Memorial Hospital, Keelung 204, Taiwan; 4 Department of Electrical Engineering, National Taiwan University, Taipei 106, Taiwan; 5 Department of Neurosurgery, New Taipei City TuCheng Hospital, New Taipei City 236, Taiwan; 6 School of Medicine, Chang Gung University, Kwei-Shan, Taoyuan 333, Taiwan

**Keywords:** surgical navigation, augmented reality, neurosurgery

## Abstract

Augmented reality surgery systems are playing an increasing role in the operating room, but applying such systems to neurosurgery presents particular challenges. In addition to using augmented reality technology to display the position of the surgical target position in 3D in real time, the application must also display the scalpel entry point and scalpel orientation, with accurate superposition on the patient. To improve the intuitiveness, efficiency, and accuracy of extra-ventricular drain surgery, this paper proposes an augmented reality surgical navigation system which accurately superimposes the surgical target position, scalpel entry point, and scalpel direction on a patient’s head and displays this data on a tablet. The accuracy of the optical measurement system (NDI Polaris Vicra) was first independently tested, and then complemented by the design of functions to help the surgeon quickly identify the surgical target position and determine the preferred entry point. A tablet PC was used to display the superimposed images of the surgical target, entry point, and scalpel on top of the patient, allowing for correct scalpel orientation. Digital imaging and communications in medicine (DICOM) results for the patient’s computed tomography were used to create a phantom and its associated AR model. This model was then imported into the application, which was then executed on the tablet. In the preoperative phase, the technician first spent 5–7 min to superimpose the virtual image of the head and the scalpel. The surgeon then took 2 min to identify the intended target position and entry point position on the tablet, which then dynamically displayed the superimposed image of the head, target position, entry point position, and scalpel (including the scalpel tip and scalpel orientation). Multiple experiments were successfully conducted on the phantom, along with six practical trials of clinical neurosurgical EVD. In the 2D-plane-superposition model, the optical measurement system (NDI Polaris Vicra) provided highly accurate visualization (2.01 ± 1.12 mm). In hospital-based clinical trials, the average technician preparation time was 6 min, while the surgeon required an average of 3.5 min to set the target and entry-point positions and accurately overlay the orientation with an NDI surgical stick. In the preparation phase, the average time required for the DICOM-formatted image processing and program import was 120 ± 30 min. The accuracy of the designed augmented reality optical surgical navigation system met clinical requirements, and can provide a visual and intuitive guide for neurosurgeons. The surgeon can use the tablet application to obtain real-time DICOM-formatted images of the patient, change the position of the surgical entry point, and instantly obtain an updated surgical path and surgical angle. The proposed design can be used as the basis for various augmented reality brain surgery navigation systems in the future.

## 1. Introduction

Surgical navigation systems. Surgical work today routinely involves interpreting images on a computer [1,2,3]. Current surgical navigation systems integrate imaging functions to help surgeons conduct preoperative simulations [4,5,6]. For example, Finke et al. [7] combined a fully electric surgical microscope with a robotic navigation system to accurately and repeatedly place the microscope without interrupting the clinical workflow. Kockro [8] combined a handheld navigation probe tracked by the tracking system with a miniature camera to observe real-time images through the miniature camera and navigate during surgery. Birth et al. [9] combined an intraoperative ultrasound probe navigation system with an online navigation waterjet dissector for clinical liver resection. However, such computer-based navigation requires surgeons to continuously shift their attention back and forth between the screen and the patient, leading to distraction and loss of focus.

AR-based surgical navigation systems. The integration of augmented reality (AR) in surgical navigation systems can enhance the intuitiveness of system use [10]. Konishi et al. [11] developed an intraoperative ultrasound (IOUS) AR navigation system to improve surgical accuracy. Pokhrel et al. [12] incorporated AR technology to effectively reduce cutting errors during knee replacement surgery by approximately 1 mm. Fotouhi et al. [13] proposed a head-mounted display (HMD)-based AR system designed to guide optimal surgical robotic arm setup.

AR-based surgical navigation system for pre-surgical simulation. Many AR applications in medical treatment are used for pre-surgical simulation and practice [14,15,16]. Chiou et al. [17] proposed an augmented reality system based on image target positioning, which can superimpose digital imaging and communications in medicine (DICOM) images over the patient’s head to provide more intuitive surgical assistance. Konishi et al. [11] performed pre-surgical computed tomography (CT) and magnetic resonance imaging (MRI) examinations on patients using body surface markers, with optical tracking used to create 3D reconstruction images which were then superimposed on the patient during subsequent surgery. Konishi et al. [18] later combined their augmented reality navigation system with a magneto-optic hybrid 3D sensor configuration to assist surgeons in performing endoscopic surgery. Okamoto et al. [19] developed a short rigid scope for use in pancreatic surgery, allowing surgeons to obtain 3D images of organs, which could then be superimposed on 3D images obtained by physicians during surgery. Tang et al. [20] applied AR technology in hepatobiliary surgery, reconstructing 3D images of liver and biliary tract structures using preoperative CT and MRI data, which were then superimposed on organs during surgery, thus enhancing the surgeon’s perception of intrahepatic structures and increasing surgical precision. Volonté et al. [21] created 3D reconstructions from CT slides, which they then projected onto the patient’s body to enhance spatial perception during surgery, and used image overlay navigation for laparoscopic operations such as cholecystectomy, abdominal exploration, distal pancreas resection, and robotic liver resection. Bourdel et al. [22] used AR technology to assist physicians in judging the location of organs, using AR to locate adenomyomas during laparoscopic examination, giving the virtual uterus a translucent appearance that allowed surgeons to better locate adenomyomas and determine uterine position.

AR-based surgical navigation system for brains neurosurgery. However, most AR surgical navigation methods are not applicable for brain surgery. Zeng et al. [23] proposed a prototype system that uses SEEG to realize see-through video augmented reality (VAR) and spatial augmented reality (SAR) in 2017. This system can help surgeons quickly and intuitively confirm registration accuracy, locate entry points, and visualize the internal anatomy in a virtual image space and the actual patient space. Léger et al. [24] studied the impact of two different types of AR-based image-guided surgery (mobile AR and desktop AR) and traditional surgical navigation on attention shifts for the specific task of craniotomy planning. In 2015, Besharati Tabrizi and Mehran [25] proposed a method for using an image projector to project an image of the patient’s skull onto the patient’s head for surgical navigation. Hou et al. [26] proposed a method to achieve augmented reality surgical navigation using a low-cost iPhone. Zhang et al. [27] developed a surgical navigation system that projects near-infrared fluorescence and ultrasound images onto Google Glass, clearly showing the tumor boundary that would otherwise be invisible to the naked eye, thus facilitating surgery. Müller et al. [28] proposed fiducial marker image navigation, using a lens to identify fiducial points and generating virtual images for percutaneous nephrolithotomy (PCNL). Prakosa et al. [29] designed AR guidance in a virtual heart to increase catheter navigation accuracy and thus affect ventricular tachycardia (VT) termination. Tu et al. [30] proposed a HoloLens-to-world registration method using an external EM tracker and a customized registration cube, improving depth perception and reducing registration.

Research Target. However, these methods do not provide a comprehensive solution for the presentation of surgical target, scalpel entry point, and scalpel orientation for neurosurgery. Therefore, we propose an AR-based optical surgical navigation system to achieve AR brain neurosurgery navigation.

## 2. Research Purpose

Neurosurgeons currently rely on DICOM-format digital images to find the operation target; determine the entry point, scalpel position, and depth; and to confirm the scalpel position and depth during the operation. Augmented reality surgical systems can help surgeons improve scalpel position and depth accuracy, while enhancing the intuitiveness and efficiency of surgery. To improve the intuitiveness, efficiency, and accuracy of EVD surgery, this paper proposes an AR-based surgical navigation system for external ventricular drain, achieving the following targets.

(1) An AR-based virtual image is superimposed on the patient’s body, thus relieving the surgeon of needing to switch views.
(2) The system directly locks the surgical target selected by the surgeon through the DICOM-formatted image.
(3) The surgeon can see the surgical target image directly superimposed on the patient’s head on a tablet computer or HMD.
(4) Once the surgeon selects the surgical entry point, it is displayed on the screen and superimposed on the patient’s head.
(5) The scalpel orientation image is displayed on the screen and superimposed on the patient’s head.
(6) The surgeon can change the surgical entry point at will, and the system will reflect such changes in real time.
(7) The scalpel entry point and direction are displayed in real time along with the surgeon’s navigation stick, providing accurate and real-time guidance.
(8) On-screen color-coded prompts ensure the surgeon uses the correct entry point and scalpel direction.
(9) The superimposed scalpel images can be displayed on the screen before and during surgery, providing surgeons with more intuitive and accurate guidance.
(10) Highly accurate image superimposition.
(11) Low pre-surgical preparation time requirements for DICOM file processing and program importing.
(12) Low preoperative preparation time in the operating room.
(13) System use does not extend the duration of traditional surgery.
(14) Clinically demonstrated feasibility and efficacy, and is ready for clinical surgical trials.

## 3. Materials, Methods and Implementations

The proposed AR-based neurosurgery navigation system uses a laptop computer (Windows 10 operating system, Intel core i7-8750H CPU @ 2.20 GHz processor, 32 G RAM memory) as a server, a tablet computer (Samsung Galaxy Tab S5e, Android 9.0 operating system and an eight-core processor) as an operating panel, and an optical measurement system (NDI Polaris Vicra) for positioning, connected by a WiFi router (ASUS RT-N13U).

The accuracy of the optical measurement system (NDI Polaris Vicra) was first verified before designing functions to allow surgeons to quickly select the desired surgical target and entry point positions, using the tablet to display superimposed images of surgical target, entry point, and scalpel to validate scalpel orientation.

**Figure 1:**
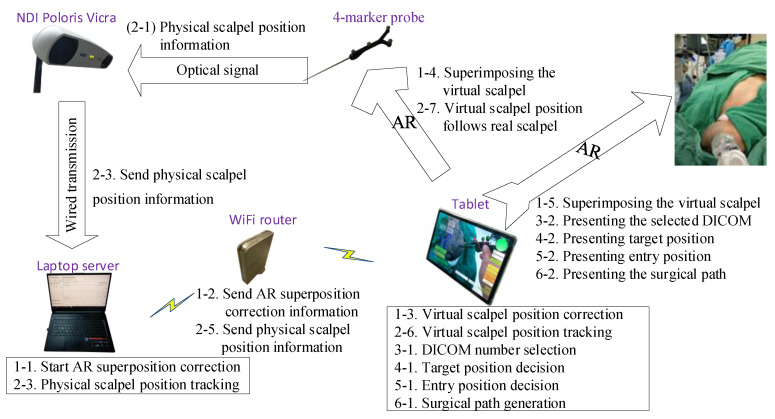
The architecture diagram.

The system can calculate corresponding 3D images using mobile devices such as AR glasses, tablet computers, and smartphones. While these three types of devices have different characteristics, this paper mainly focuses on tablet computer applications. Table 1 defines the symbols and parameters used in the proposed method, and Figure 1 shows the overall architecture diagram. The detailed implementation and operation are described in the following steps.

### 3.1. AR Model Creation

**Figure 2:**
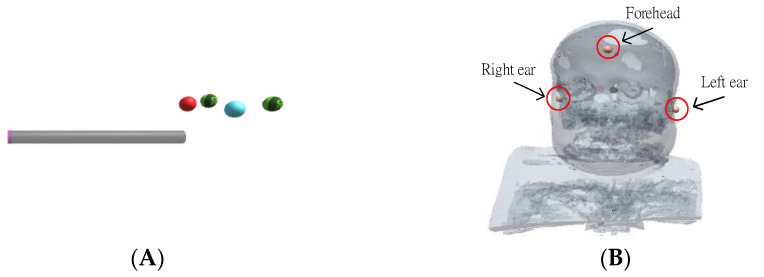
4-marker scalpel model and AR scalp model and markers. (**A**) 4-marker scalpel model. (**B**) AR scalp model and markers.

A 4-marker scalpel model (Figure 2A) was created in Unity (a cross-platform game engine developed by Unity Technologies), using the patient’s CT or MRI DICOM data to make an AR scalp model (Figure 2B through the Avizo image analysis software, placing markers on the ears and top of the AR scalp model (Figure 2B).

### 3.2. AR Superimposition Accuracy

**Figure 3:**
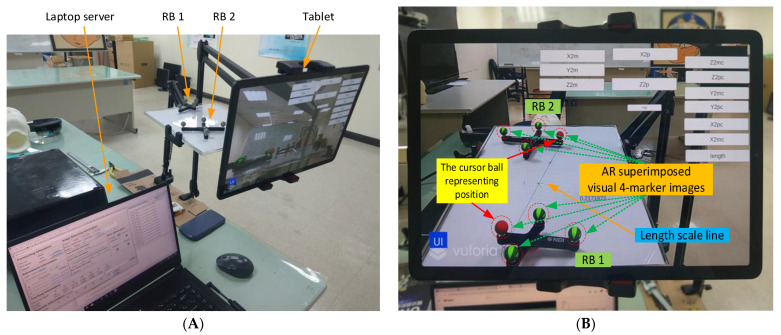

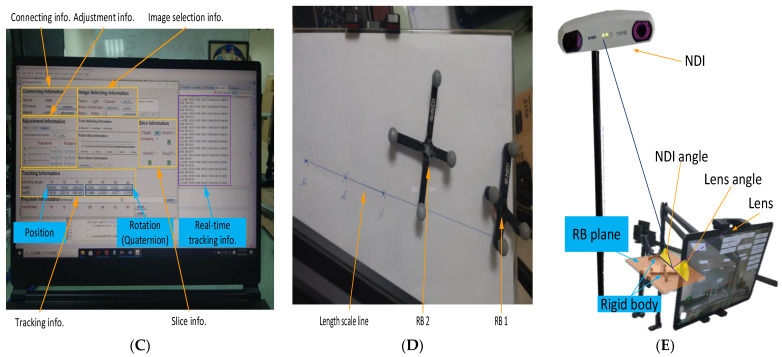
AR superimposition accuracy test. (**A**) Overall hardware implementation. (**B**) Tablet display. (**C**) Server interface. (**D**) Different placement of RBs. (**E**) NDI and lens angles.

Manual AR superimposition in the preoperative phase may produce errors. To understand and minimize such errors, we evaluated the impact of each component on AR accuracy, including the laptop server, the NDI Polaris Vicra, the tablet, the wireless router, and two rigid bodies. (Figure 3A).

First, two sets of AR 4-marker images were superimposed on two sets of 4-marker rigid bodies (Figure 3B). The distance between the two sets of 4-marker rigid bodies was calculated according to the coordinate data from NDI Polaris Vicra (Figure 3C), and the distance between the two sets of AR 4-marker images was calculated according to the coordinate data using Unity. The distance differences were then compared using the same method to modify the distances and angles (Figure 3D) for error testing.

Each rigid body has four cursor balls, one of which indicates the body’s position, here identified as a red virtual AR ball (see Figure 3B). The position of this ball in the optical positioning world represents the coordinate data (position) of the rigid body sent by the NDI Polaris Vicra. In the virtual world, this position represents the position of the red AR virtual image ball displayed in Unity. The two rigid bodies were positioned on a scaled line (respectively 1, 2, 3, 4, and 5 cm) to measure (1) their actual distance, (2) the distance calculated from the NDI Polaris Vicra data, and (3) the distance calculated from the position displayed by Unity. These three distance datapoints were then used to calculate the accuracy and error values. The impact of viewing angle on accuracy was assessed using angles of 0 degrees, 45 degrees, and 90 degrees (Figure 3D).

Figure 3C shows the user interface with eight information blocks. The connection information block is used to connect to NDI Polaris Vicra via a wired USB cable and to connect to a tablet via the WiFi router. The adjustment information block is used to adjust the position and rotation of the AR virtual image. The image selection information block is used to select specific parts of the head in the AR virtual image, such as face, specific bones, brain, tumors, etc. The tracking information block is used to obtain the rigid body coordinate data sent by the NDI Polaris Vicra. This block contains the three-coordinate positioning information and the rotation of the quaternion. In addition to calculating the relative position of the two rigid bodies, the degree of rotation of each rigid body must also be used to calculate the relative angle between the two rigid bodies. The slice information block is used to select slices of various DICOM orientations to display the AR virtual images, including the XY, YZ, and XZ planes.

The three remaining blocks (tools selection, future slice, and error detection) are rarely used. They are used to select various tools (such as scalpel or ultrasound), to display the extension of the scalpel image in the AR virtual image, and to facilitate error detection within the program. Aside from the user interface, the programming interface continuously collects tracking information (including current and past tracking information) in real time.

As shown in Figure 3E, the lens/NDI angle is the angle between the RB plane (i.e., XZ plane) and the extension line of the tablet lens/NDI positioning device. Test results found that the distance error of the NDI positioning device is smallest when the NDI angle is 90 degrees. Similarly, after adding the AR function, the distance error of Unity is smallest when the lens angle is 90 degrees. However, the NDI angle and the lens angle cannot be 90 degrees at the same time. We also found that when the NDI angle changes, the effect of the distance error of the NDI positioning device is smaller than that of the Unity distance error when the lens angle changes. Therefore, we tested the comprehensive error of NDI and Unity by changing the RB plane (Figure 3E). We can see that the closer the lens angle is to 90 degrees, the farther the NDI angle is from 90 degrees. However, when the lens angle is 90 degrees, we can obtain the smallest distance error and the highest stability with the best overall performance due to the reduced influence of the NDI angle change.

**Figure 4:**
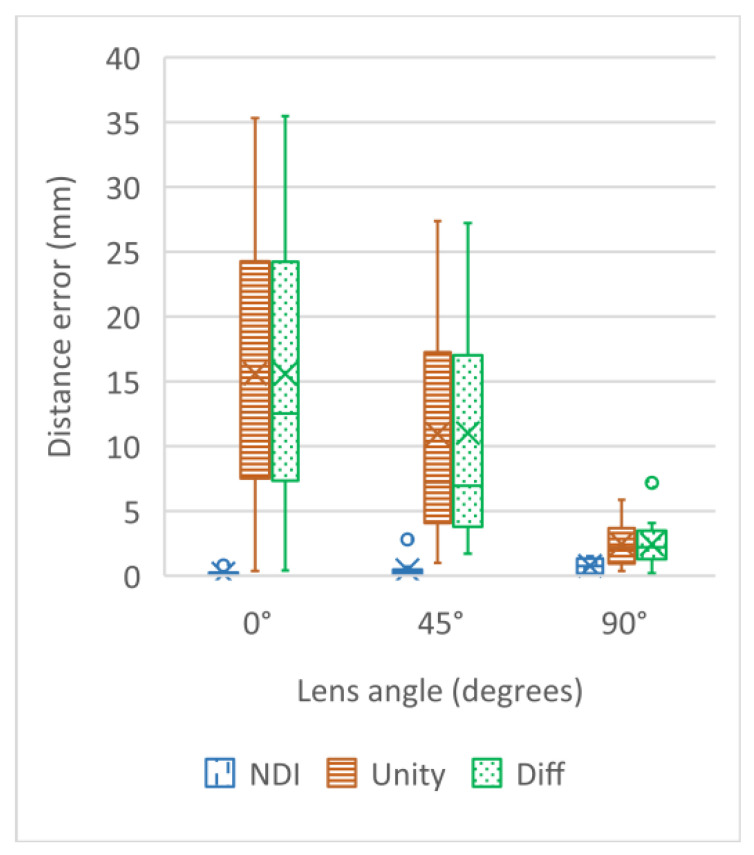
Mean and standard deviation of distance error (mm).

The mean and the standard deviation of the distance errors are shown in Figure 4 and Table 2, where lens angle means the angle between the 4-marker-rigid-bodies plane angle and the tablet–PC–camera–lens photography angle (Figure 3E). NDI distance and Unity distance stand for the error (mean or variance) between the “actual distance” and the “calculated distance”, which are respectively calculated based on the values of the two sets of rigid bodies displayed by the NDI Polaris Vicra, and based on the two sets of AR 4-marker images displayed by Unity. Difference means the difference between each NDI distance and the Unity distance. NDI distance works best with a lens angle of 0 degrees, while Unity distance works best with a lens angle of 90 degrees. All things considered, this AR system works best with a 90 degree lens angle. From the result, the reliable lens angle is 90 ± 10 degrees. A lens angle of approximately 90 degrees minimizes the AR superimposition error and maximizes data stability, with a mean error of 2.01 ± 1.12 mm. (Note: Abdoh’s review article, [31], found considerable variation in intracranial catheter length from 5 to 7 cm.)

### 3.3. Laboratory Simulated Clinical Trials

**Figure 5:**
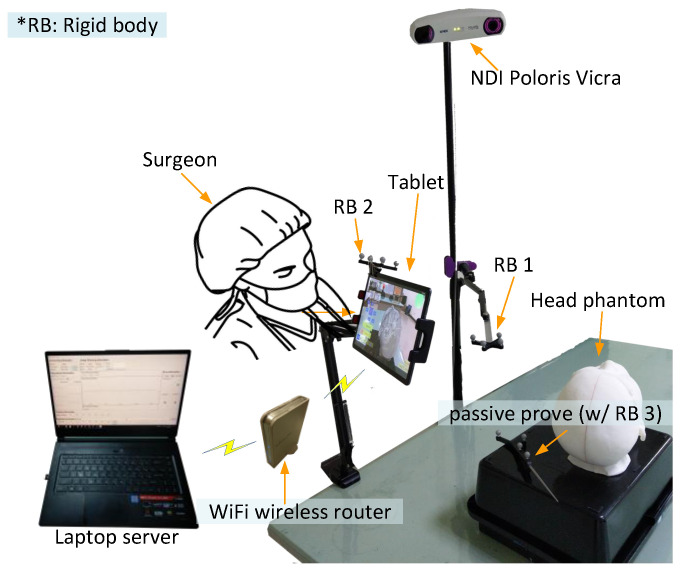
Laboratory simulated clinical trials.

Simulated experiments were used to revise various parameters. DICOM data for selected patients was used to create a high-accuracy head phantom (Figure 5).

The proposed system was developed in two versions, with three and two rigid bodies. In the three-rigid-bodies version, the three rigid bodies were located on the navigation stick, the bed, and the top of the tablet, used in positioning the scalpel, the head, and the tablet (i.e., the camera), respectively. The two-rigid-bodies version did not include the tablet. The three-rigid-bodies version tracks real-time movement of the tablet, but the position and rotation information of these three rigid bodies must be calculated in real time, along with their relative positions and angles, resulting in occasional screen lag. The 2-rigid-bodies version is thus more stable, but the tablet must remain in a static position. Otherwise, AR superimposition must be used to recalibrate relative positioning information.

**Figure 6:**
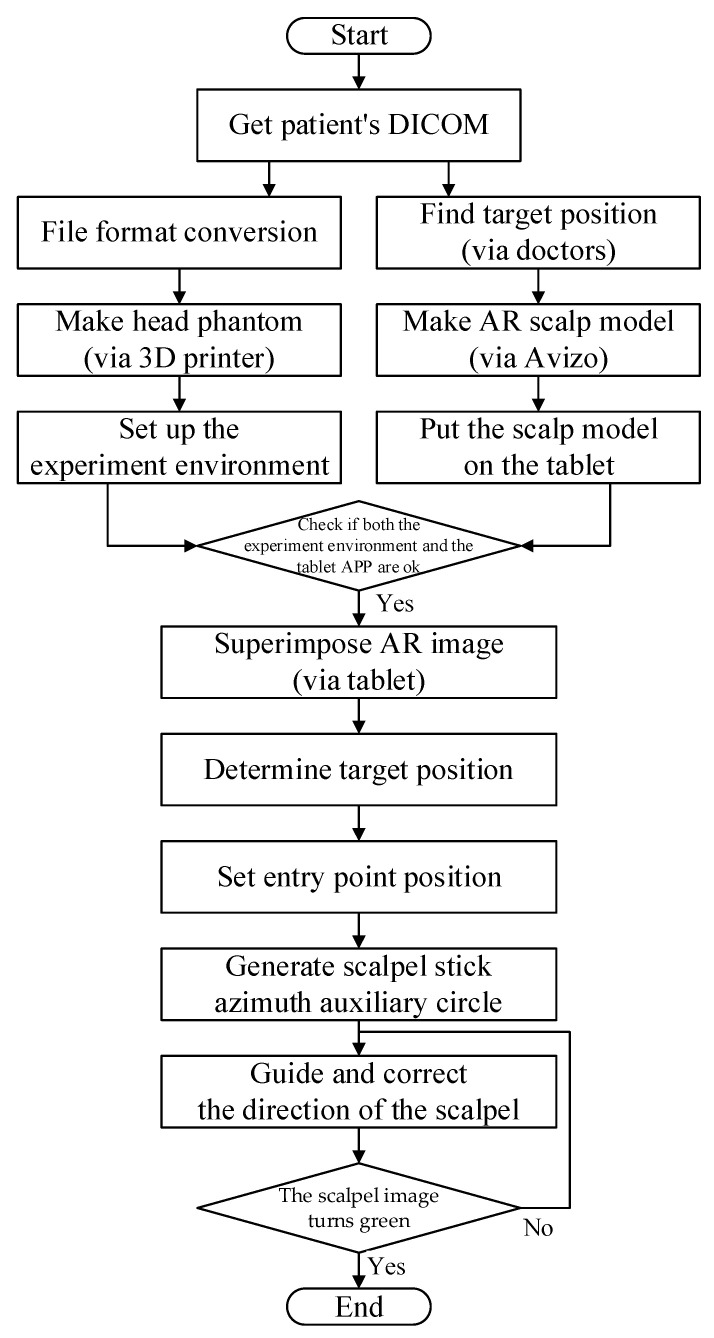
Laboratory simulation flow chart.

**Figure 7:**
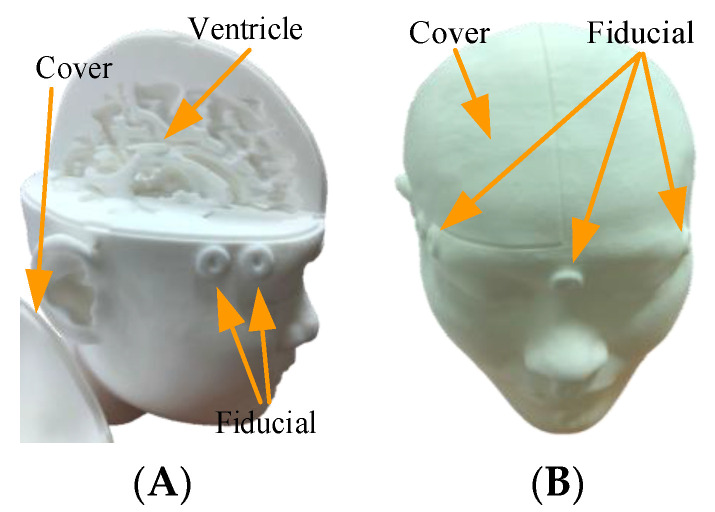
Patient-specific head phantom. (**A**) Head phantom with the ventricle. (**B**) Head phantom of combined appearance.

Laboratory tests were conducted to assess hardware and software pre-processing performance, followed by AR overlay experiments (see Figure 6). In the hardware tests, the patient’s DICOM data with 147 slices, each with a thickness of 2 mm and a file size 74.0 MB, were converted into a 3D printer format to create a patient-specific head phantom model, including the patient’s ventricle and a head cover. The model was then printed using white nylon (see Figure 7), and placed in the experimental environment. The software test asked the surgeon to identify the target position in DICOM, after which Avizo was used to make an AR scalp model from the DICOM data.

This target information can optionally be manually set at the target position found by the surgeon. The AR scalp model is then loaded into the tablet APP.

Software and hardware pre-processing was followed by AR-based virtual image overlay experiments. The steps in Section 3 were followed to determine the target position of the patient, which was then displayed on the tablet so that it overlapped the patient’s head. After the entry point position has been determined, the APP automatically generates a scalpel stick azimuth auxiliary circle on the upper right of the tablet. The surgeon then corrects the scalpel direction until the scalpel image turns green, indicating correct direction.

### 3.4. Experimental Setup

**Figure 8:**
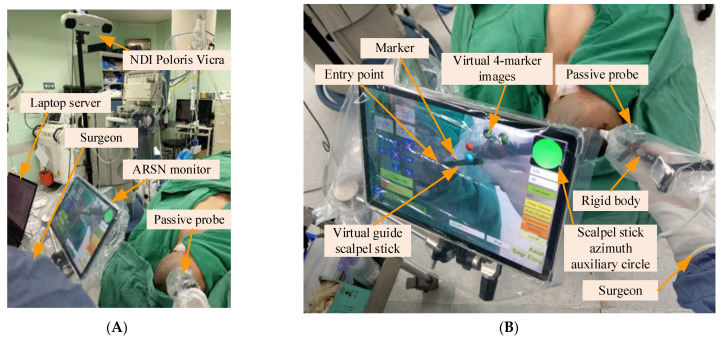
Clinical system architecture and tablet close-up. (**A**) Overall structure. (**B**) Tablet close-up.

The system hardware, including the laptop server, the optical measurement system (i.e., NDI Polaris Vicra), the tablet computer, the wireless router, and the 4-marker probe (i.e., scalpel stick) were arrayed around an operating bed (Figure 8).

### 3.5. AR-Based Virtual Image Superimposition

**Figure 9:**
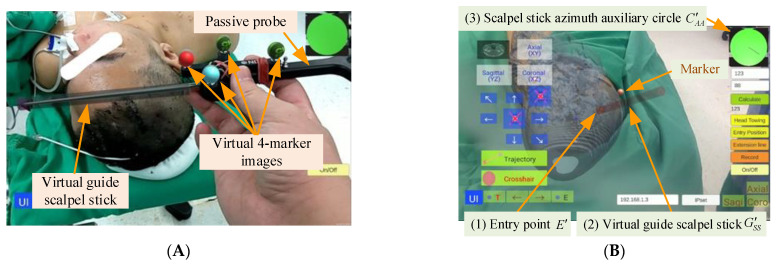
AR scalpel superimposition. (**A**) Superimposed 4-marker images and scalpel stick. (**B**) Superimposed AR head model and guide scalpel stick.

In the preoperative phase, a technician took 5–7 min to superimpose the virtual head image and scalpel. The 4-marker scalpel model was then superimposed on the 4-marker probe (Figure 9A), and then the AR head model was superimposed on the patient’s head based on the three markers (right and left ears and top of head) (Figure 9B).

### 3.6. Target Position Determination

**Figure 10:**
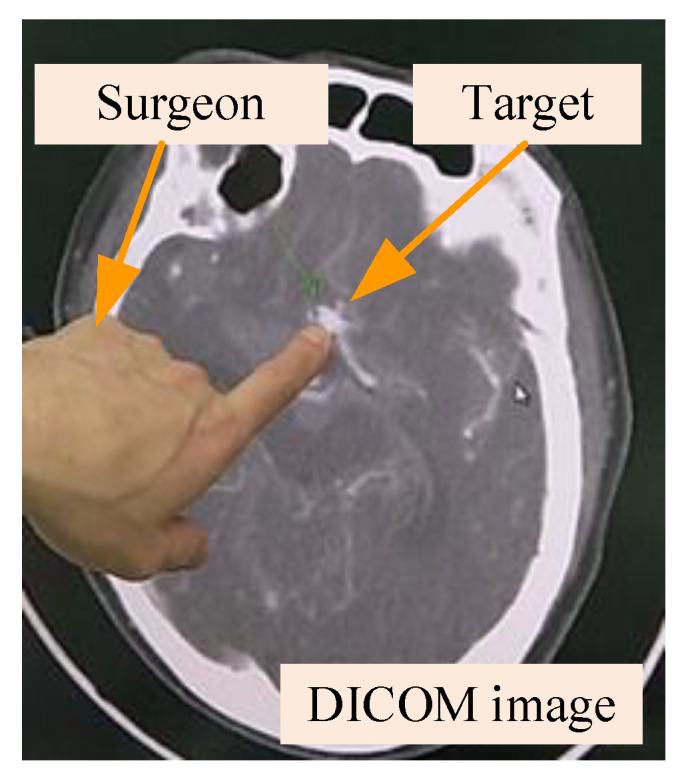
Manually identify the target position.

**Figure 11:**
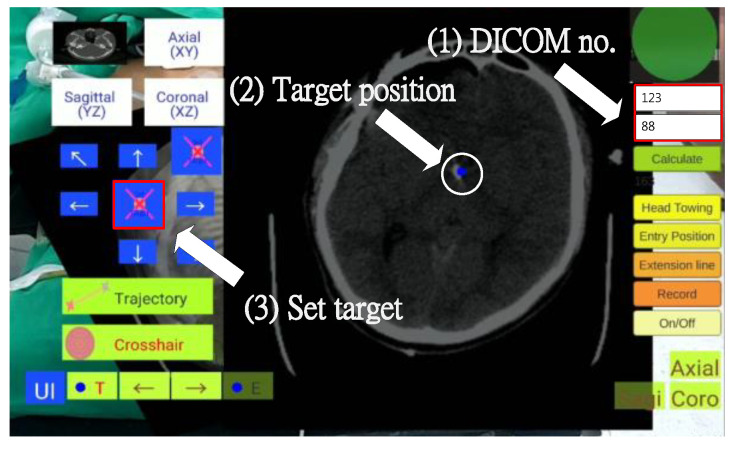
Find and set target position.

In this step, the surgeon required about one minute to establish the target position, observing the DICOM-formatted images on a computer or directly on the tablet PC to identify the surgical target and obtain its DICOM page number (Figure 10). The surgeon then input the DICOM page number and determined the target position by tapping the tablet screen (displaying a ball), and clicked the “Set Target” button to complete the target positioning (Figure 11).

### 3.7. Entry Point Position Setting

**Figure 12:**
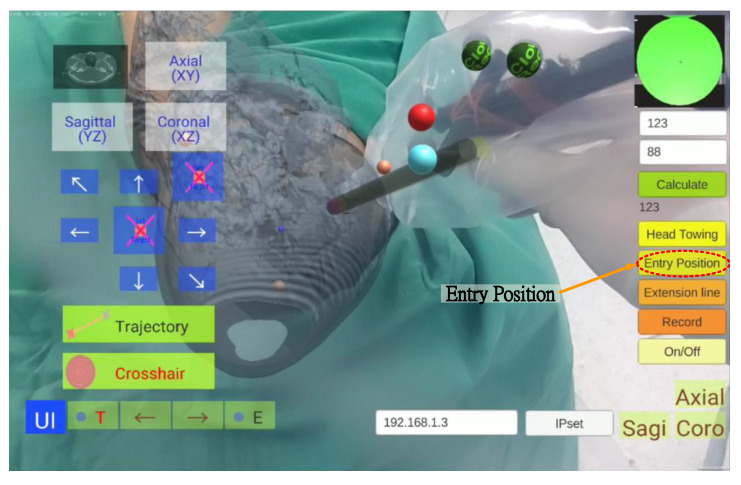
Entry point position setting.

In this step, the surgeon selected an entry point position and set it on the tablet, an operation that took approximately one minute. The surgeon clicked the “Entry Position” button to superimpose the AR entry point image over the scalpel tip (Figure 12). When the surgeon moved the scalpel tip to determine the entry point, he pressed the “Entry Position” button again to complete the setting.

### 3.8. Scalpel Orientation Guidance and Correction

In this step, the tablet dynamically displays the superimposed image of the scalp, target position, entry point position, and scalpel (including the scalpel tip and scalpel spatial direction calibrator).

**Figure 13:**
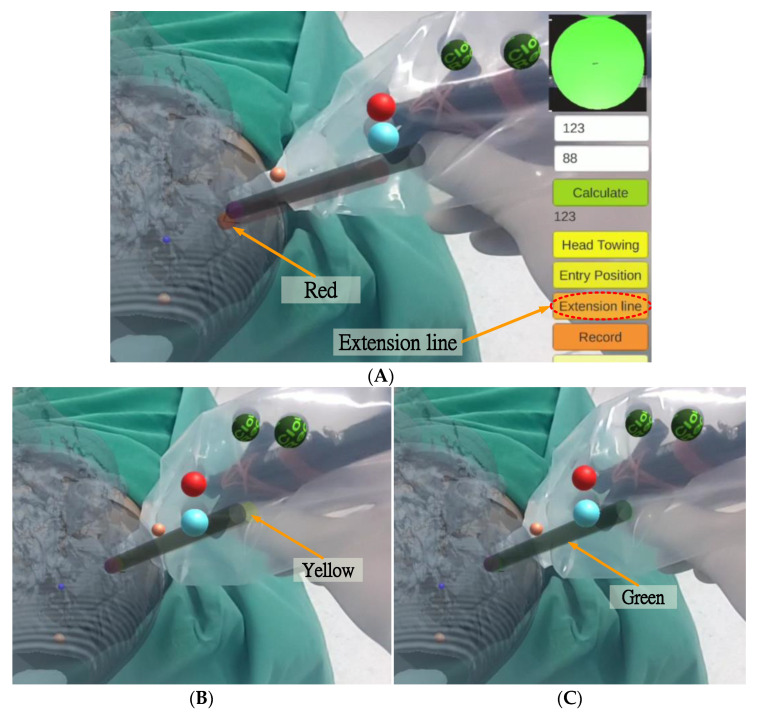
Extension line and three-color scalpel stick. (**A**) Extension line and red scalpel stick. (**B**) Yellow scalpel stick. (**C**) Green scalpel stick.

After setting the entry point position, the surgeon pressed the “Extension line” button, and a red scalpel stick virtual image (the virtual guide scalpel stick ${G^{\prime }}_{SS}$) appeared superimposed over the scalp as a scalpel path extension bar, guiding the surgeon vis-à-vis the scalpel position and angle (Figure 13A). The surgeon can fine-tune the scalpel position and angle based on the color of the virtual scalpel stick and the orientation calibrator (a green circle in the upper right corner).

The color change rule of the scalpel stick virtual image is as follows:

$$\mathrm{color}=\{\begin{array}{l} \mathrm{Red},\;\mathrm{if}\;d(p_{T}({S^{\prime }}_{SM}),p(E^{\prime }))>2\;\mathrm{mm}\\ \mathrm{Yellow},\;\mathrm{if}\;d(p_{T}({S^{\prime }}_{SM}),p(E^{\prime }))\leq 2\;\mathrm{mm},\;∠(v({S^{\prime }}_{SM}),v({L^{\prime }}_{A}))>1.5^{o}\\ \mathrm{Green},\;\mathrm{if}\;d(p_{T}({S^{\prime }}_{SM}),p(E^{\prime }))\leq 2\;\mathrm{mm},\;∠(v({S^{\prime }}_{SM}),v({L^{\prime }}_{A}))\leq 1.5^{o} \end{array},$$

where

(1) $$d(p1,p2)=\sqrt{{\left(p1_{x}-p2_{x}\right)}^{2}+{\left(p1_{y}-p2_{y}\right)}^{2}+{\left(p1_{z}-p2_{z}\right)}^{2}}$$

(2) $$∠(v({S^{\prime }}_{SM}),v({L^{\prime }}_{A}))=a\cos(\frac{v({S^{\prime }}_{SM})\cdot v({L^{\prime }}_{A})}{\left|v({S^{\prime }}_{SM})\right|\cdot \left|v({L^{\prime }}_{A})\right|})$$

$E^{\prime }$, ${L^{\prime }}_{A}$, and ${S^{\prime }}_{SM}$ represent the virtual entry point, the virtual auxiliary line, and the virtual stick of 4-marker scalpel model, respectively (Figure 2A); p(x) and v(x) are the position and the vector of x, respectively; $p_{T}(x)$ is the tip point of x, and $\left(A_{x},A_{y},A_{z}\right)$ are the coordinates of A.

If the distance between the tip position of the scalpel image and the actual entry point is less than 2 mm and the angle between the scalpel and the scalpel stick virtual image is greater than 1.5 degrees, the scalpel image will turn yellow (Figure 13B), indicating that the position of the scalpel tip is correct but the angle is incorrect. If the distance between the tip position of the scalpel image and the actual entry point is less than 2 mm and the angle between the scalpel and the scalpel stick virtual image is less than 1.5 degrees, the scalpel image will turn green (Figure 13C), indicating that both the position of the scalpel tip and the angle are correct.

### 3.9. Scalpel Stick Azimuth Auxiliary Circle

In the upper right corner of the UI, there is a scalpel stick azimuth auxiliary circle ${C^{\prime }}_{AA}$, which can assist surgeons in correcting the orientation of the surgical scalpel stick (Figure 9). The surgeon can infer whether the angle of the real scalpel stick is consistent with the angle of the virtual guide scalpel stick according to the length and the angle of the line in the circle, and adjust the orientation accordingly.

The shorter the length of this line (i.e., closer to a point), the smaller the angle difference. The line’s deviation direction stands for the real scalpel stick’s direction of deviation. The technical steps of ${C^{\prime }}_{AA}$ creation are as follows.

The system first creates a virtual auxiliary circle ${C^{\prime }}_{A}$ in Unity and sets its position $p({C^{\prime }}_{A})$ at the position of the virtual entry point $E^{\prime }$:

(3) $$p({C^{\prime }}_{A})\leftarrow p(E^{\prime })$$

It then sets the rotation of ${C^{\prime }}_{A}$ as orthogonal to the virtual guide scalpel stick ${G^{\prime }}_{SS}$:

(4) $$r({C^{\prime }}_{A})\leftarrow r_{⊥}({G^{\prime }}_{SS})$$

where p(x) and r(x) stand for the position and rotation of x, respectively, and $r_{⊥}(x)$ is an orthogonal rotation such that $r_{⊥}(x)$ and r(x) are orthogonal.

Then the system then creates a virtual auxiliary line, ${L^{\prime }}_{A}$, and sets the start position of this line at the position of ${G^{\prime }}_{SS}$:

(5) $$p_{0}({L^{\prime }}_{A})\leftarrow p({G^{\prime }}_{SS})$$

The rotation of ${L^{\prime }}_{A}$ is equal to that of ${S^{\prime }}_{SM}$:

(6) $$r({L^{\prime }}_{A})\leftarrow r({S^{\prime }}_{SM})$$

The length of this line is the radius of ${C^{\prime }}_{A}$:

(7) $$length({L^{\prime }}_{A})\leftarrow radius({C^{\prime }}_{A})$$

where $p_{0}(l)$ and length(l) respectively stand for the start position and the length of the line l, and ${S^{\prime }}_{SM}$ is the virtual stick of the 4-marker scalpel model (Figure 2A). Finally, the system creates a virtual scalpel stick azimuth auxiliary line ${L^{\prime }}_{A}^{\;vp}$:

(8) $${L^{\prime }}_{A}^{\;vp}=\frac{{L^{\prime }}_{A}\cdot {L^{\prime }}_{B}}{{\left|{L^{\prime }}_{B}\right|}^{2}}\cdot {L^{\prime }}_{B}$$

and produces the scalpel stick azimuth auxiliary circle ${C^{\prime }}_{AA}$ and the scalpel stick azimuth auxiliary line ${L^{\prime }}_{AA}$:

(9) $$\left({C^{\prime }}_{AA},{L^{\prime }}_{AA})\leftarrow ({C^{\prime }}_{A},{L^{\prime }}_{A}^{\;vp}\right)$$

The system then displays ${C^{\prime }}_{AA}$ and ${L^{\prime }}_{AA}$ in the upper right of the UI, where ${L^{\prime }}_{A}^{\;vp}$ is a vector projection of ${L^{\prime }}_{A}$ onto ${C^{\prime }}_{A}$, line ${L^{\prime }}_{B}$ is the normal vector of line ${L^{\prime }}_{A}$ in circle ${C^{\prime }}_{A}$, and (${C^{\prime }}_{AA}$, ${L^{\prime }}_{AA}$) is identical to (${C^{\prime }}_{A}$, ${L^{\prime }}_{A}^{\;vp}$).

## 4. Results

This study proposes an AR-based optical surgical navigation system for use in the preoperative stage of EVD surgery by accurately superimposing virtual images of the surgical target position, scalpel entry point, and scalpel direction over the patient’s head. This superimposition is displayed on a tablet, where the color of the virtual scalpel stick and an azimuth auxiliary circle increase the accuracy, intuitiveness, and efficiency of EVD surgery. The resulting accuracy is within 2.01 ± 1.12 mm, presenting a significant improvement over the optical positioning navigation (18.8 ± 8.56 mm) proposed by Ieiri et al. [32], the mobile AR for percutaneous nephrolithotomy (7.9 mm) proposed by Müller et al. [28], the phantom and porcine model AR evaluation (2.8 ± 2.7 and 3.52 ± 3.00 mm proposed by Kenngott et al. [33], the AR imaging for endonasal skull base surgery (2.8 ± 2.7 mm) proposed by Lai et al. [34], and the image positioning navigation (2.5 mm) proposed by Deng et al. [35]. Table 3 shows that the proposed optical positioning method provided the best results in terms of average accuracy and standard deviation.

In addition, a total of four clinical trials of external ventricular drain (EVD) surgery were performed as follows:

(1) DICOM acquisition. Patient CT DICOM data was obtained from Keelung Chang Gung Memorial Hospital.
(2) AR scalp image production. Avizo was used to create a 3D virtual scalp image from the patient DICOM data.
(3) Data import. 3D virtual scalp images and patient DICOM data were imported into Unity, and used to produce and update the tablet PC APP.
(4) APP test and surgery simulation. Following APP update, simulated surgery was conducted to ensure the correctness of the APP.
(5) Clinical trials. Clinical trials were conducted at Keelung Chang Gung Memorial Hospital.

Steps (1) to (4) of the preparation stage required an average of 2 ± 0.5 h to complete. Step (5), the preoperative stage (including virtual image superimposition, target position determination, entry point position setting, and scalpel orientation guidance and correction), required 10 ± 2 min on average.

## 5. Discussion

A good surgical navigation system needs the following four properties: convenient instruments, rapid DICOM data processing, instant operation, and high precision.

The method proposed by Chiou et al. [17] enables the instant display of DICOM-formatted images superimposed on the patient’s head. However, their method is only a concept, and can only place the identification map under the patient’s head. They do not mention superimposition accuracy or the method used to superimpose the virtual image.

Kenngott et al. [33] performed three AR evaluations: phantom model evaluation (with a mean reprojection error of 2.8 ± 2.7 mm), porcine model evaluation (with a mean reprojection error of 3.52 ± 3.00 mm), and human feasibility test. Although their system outperforms other AR-like systems in terms of accuracy, they provided no accuracy data for human testing.

Tabrizi and Mehran [25] proposed a method which uses an image projector to create augmented reality by projecting an image of the patient’s skull on the patient’s actual head, thereby enhancing surgical navigation. However, their method cannot display the scalpel’s relative position in the augmented reality, and also cannot display detailed brain tissue structures or the corresponding position and angle of the CT image.

Hou et al. [26] implemented augmented reality surgical navigation using a low-cost iPhone. However, this approach only provides AR images from specific angles, making it poorly suited to actual surgical conditions.

Zhang et al. [27] developed a surgical navigation system using near-infrared fluorescence and ultrasound images that can be automatically displayed on Google Glass, clearly showing tumor boundaries that are invisible to the naked eye. However, their current implementation still has some significant shortcomings and limitations, particularly related to Google Glass’s short battery life, its tendency to overheat, its limited field of view, and limited focal length. Another limitation is that the ICG used in fluorescent imaging is not a tumor-specific contrast agent, so it cannot be used in tumor-specific clinical applications.

The fiducial marker image navigation proposed by Müller et al. [28] uses lenses to identify fiducials and generate virtual images for percutaneous nephrolithotomy (PCNL). However, in addition to the time-consuming pre-surgical placement of reference points (99 s), the average error of 2.5 mm is not conducive to neurosurgical procedures (such as EVD).

In summary, previously proposed methods do not present a comprehensive solution for the accurate guidance for surgical targets, scalpel entry points, and scalpel orientation in brain surgery, and the proposed approach seeks to address these shortcomings.

To maximize user convenience, the proposed system uses a tablet PC as the primary AR device, ensuring ease of portability. DICOM data processing takes about two hours to complete the system update. Surgeons can use the proposed system before and during surgery for real time guidance for surgical target, entry point, and scalpel path. Finally, in terms of precision, the proposed system has an average spatial error is 2.01 ± 1.12 mm, a considerable improvement on many previous methods.

## 6. Conclusions

This paper proposes an AR-based surgical navigation system, which superimposes a virtual image over the patient, providing surgeons with more intuitive and accurate operations without the need to shift views. The system directly locks on the surgical target selected by the surgeon, with images of the surgical target, the scalpel entry point, and the scalpel orientation all displayed on the screen and superimposed on the patient’s head, providing the surgeon with a highly accurate and intuitive view which changes in real time in response to the surgeon’s actions while providing visual prompts to assist the surgeon in optimizing scalpel entry point and direction.

In addition, an on-screen image of the scalpel entry point and orientation guides the surgeon in real-time, with guidance provided by color-coded on-screen prompts and an azimuth auxiliary circle. In the DICOM-formatted image display mode, the DICOM-formatted image of the operation target position can be displayed and accurately superimposed on the correct position (of the virtual head). When the physician switches or moves the DICOM position, the on-screen DICOM-formatted image adjusts in real time, shifting to the new relative position.

The proposed method outperforms other existing approaches in terms of mean precision (2.01 ± 1.12 mm) and standard deviation. The preparation time required before each procedure is within acceptable limits, with an average of 120 ± 30 min needed for DICOM file processing and program importing, while preoperative preparation only required six minutes on average during hospital clinical trials, and only 3.5 min were needed on average for the surgeon to set the target and entry point location and accurately identify the direction with the surgical stick.

Future work will seek to further reduce superposition error, along with time required for the preparation and preoperative stages, thus improving the clinical utility of the proposed system.

## Figures and Tables

**Table 1:** Notations.

Notation	Definition
E	Entry point
$E^{\prime }$	Virtual entry point
${C^{\prime }}_{A}$	Virtual auxiliary circle of $C_{A}$
p(x)	Position of a virtual point x
v(x)	Vector of x
$p_{T}(x)$	Tip point of x
$\left(A_{x},A_{y},A_{z}\right)$	Coordinate of A
r(x)	Rotation of a virtual point x
$r_{⊥}(x)$	Orthogonal rotation such that $r_{⊥}(x)$ and r(x) are orthogonal
	Virtual auxiliary line
${G^{\prime }}_{SS}$	Virtual guide scalpel stick
${S^{\prime }}_{SM}$	Virtual stick of 4-marker scalpel model
$p_{0}(l)$	Start position of the line l
length(l)	Length of the line l
${L^{\prime }}_{A}^{\;vp}$	Virtual scalpel stick azimuth auxiliary line
${C^{\prime }}_{AA}$	Scalpel stick azimuth auxiliary circle
${L^{\prime }}_{AA}$	Scalpel stick azimuth auxiliary line

**Table 2:** Mean and standard deviation of distance error (mm).

Lens Angle	NDI Distance	Unity Distance	Difference
0°	0.19 ± 0.20	15.61 ± 10.73	15.59 ± 10.76
45°	0.46 ± 0.63	10.94 ± 8.13	11.01 ± 8.34
90°	0.77 ± 0.59	2.46 ± 1.72	2.01 ± 1.12

**Table 3:** Comparison of related works.

	Mean ± Std (mm)	Surgical Goals
Proposed	2.01 ± 1.12	External ventricular drain
[33]-Phantom	2.8 ± 2.7	Pilot study
[33]-Porcine	3.52 ± 3.00	Pilot study
[28]	7.9	Percutaneous nephrolithotomy
[35]	2.5	Euronavigation
[34]	2.8 ± 2.7	Endonasal skull base surgery
[32]	18.8 ± 8.56	Laparoscopic splenectomy

## Data Availability

The statistical data presented in this study are available in Table 2. The datasets used and/or analyzed during the current study are available from the corresponding author upon request. These data are not publicly available due to privacy concerns and ethical reasons.
